# Comparison of the Effects of Hydralazine and Labetalol on Intracranial Pressure When Used for Blood Pressure Control in Patients With Intracranial Hemorrhage: A Retrospective Study

**DOI:** 10.7759/cureus.60914

**Published:** 2024-05-23

**Authors:** Elizabeth Timanus, Allison R Lauver, Lillianne D Stanitsas, Tracy Rock, Barbara M Hileman, Elisha A Chance

**Affiliations:** 1 Pharmacy, Mercy Health St. Elizabeth Youngstown Hospital, Youngstown, USA; 2 Trauma, Critical Care, and General Surgery Services, Mercy Health St. Elizabeth Youngstown Hospital, Youngstown, USA; 3 Trauma and Neuroscience Research, Mercy Health St. Elizabeth Youngstown Hospital, Youngstown, USA

**Keywords:** blood pressure, intracranial pressure, intracranial hemorrhage, labetalol, hydralazine

## Abstract

Background

Recommendations on optimal agents to manage blood pressure (BP) in patients with an intracranial hemorrhage (ICH) are lacking. A case series suggests that hydralazine can cause intracranial pressure (ICP) elevation in an ICH. The purpose of this study was to compare the effects of intravenous (IV) hydralazine to IV labetalol on ICP in patients with ICH.

Materials and methods

A retrospective chart review from September 2015 to September 2021 on adults admitted to a level I trauma center with ICH, requiring an external ventricular drain or ICP monitor, and pharmacologic intervention with IV hydralazine or IV labetalol. ICP measurements and clinical interventions 0-80 minutes prior to and after medication administration were compared. Data points were excluded if multiple antihypertensive agents were administered.

Results

A total of 27 patients were included (three received only hydralazine, 13 only labetalol, and 11 both). Twenty-seven doses of hydralazine and 115 doses of labetalol were compared. There was no significant difference in mean ICP 0-80 minutes following hydralazine and labetalol administration (p = 0.283). Of the hydralazine doses, 29.6% received intervention for elevated ICP, while 25.2% of labetalol doses received intervention (p = 0.633). Hydralazine patients received m = 0.56 interventions for ICP, and labetalol patients received m = 0.36 interventions (p = 0.223). Of the patients that required intervention for ICP management, hydralazine patients required m = 1.88 interventions, while labetalol patients required m = 1.41 interventions (p = 0.115).

Conclusion

There was no significant difference in mean ICP at 0-80 minutes following administration of hydralazine or labetalol. There was also no significant difference in interventions required for elevated ICP management between groups. Larger studies are needed to confirm these findings.

## Introduction

The medical management of patients following intracranial hemorrhage (ICH) either due to traumatic brain injury or hemorrhagic stroke focuses on the prevention of secondary injury. An overriding principle for the prevention of secondary injury is maintaining cerebral perfusion pressure (CPP), which is representative of cerebral blood flow as well as oxygen and nutrient delivery to the injured tissue [1]. The 2016 Brain Trauma Foundation guidelines recommend targeting a CPP between 60 and 70 mmHg for survival and favorable outcomes [2]. CPP can be described as a relationship between the mean arterial pressure (MAP) and intracranial pressure (ICP) (CPP = MAP - ICP). Therefore, target CPP can be achieved by manipulating the MAP and ICP with clinical interventions [2].

The Monro-Kellie Hypothesis describes the cranium as a fixed space and ICP as a function of the volume of the cerebrospinal fluid (CSF), brain parenchyma, and blood. If the volume of any of these three components exceeds that of the fixed space of the cranium, the ICP will rise [1]. Therefore, many clinical interventions decrease ICP by decreasing cerebral edema and brain parenchyma volume. The Brain Trauma Foundation guidelines recommend monitoring ICP in patients who have a post-resuscitation Glasgow Coma Scale (GCS) score of 3-8 and an abnormal computed tomography (CT) scan or for those with a similar severity and normal CT scan who are older than 40 years, posturing, or hypotensive [2]. Placement of an external ventricular drain allows for both direct measurement of the ICP and diversion of the CSF to decrease pressure.

In patients with ICH, the management of blood pressure (BP) is important to limit bleeding, and the associated increase in intracranial blood volume and ICP. The 2015 American Heart Association (AHA) and American Stroke Association (ASA) Guidelines for the management of spontaneous intracerebral hemorrhage recommend acute lowering of systolic BP (SBP) to 140 mmHg for ICH patients presenting with SBP between 150 and 220 mmHg and without contraindications to acute BP treatment [3]. BP should be controlled in all ICH patients for the prevention of recurrent ICH [3]. The AHA/ASA Guidelines were updated in 2022 and add to the recommendation of acute BP lowering to a target of 140 mmHg with a goal of maintaining a range between 130 and 150 mmHg systolic [4]. While more recent recommendations are available this research was initiated and designed in 2021 with the 2015 AHA/ASA guidelines in mind. The evidence for BP lowering in the setting of ICH is well established; however, guidance on antihypertensive agent selection is less defined.

The administration of as needed doses of intravenous (IV) labetalol and hydralazine for acute BP control is common practice in this clinical setting with labetalol and hydralazine routinely utilized. Labetalol is a nonselective beta-adrenergic receptor blocker with additional alpha-1 adrenergic receptor blockade with a peak effect within five to 15 minutes [5]. This agent is recommended in clinical guidelines as a safe option for BP management in patients with ICH and is often considered a first-line option but is avoided in patients unable to tolerate a decrease in heart rate or at risk for bradycardia [4]. Therefore, hydralazine is often used as an alternative; however, the available literature on the use of IV hydralazine is more limited [3]. Mechanistically, hydralazine exerts its antihypertensive properties through direct vasodilation of arterioles and has an onset of action anywhere from 10 to 80 minutes when administered intravenously [6]. Since it reduces peripheral vascular resistance, it can lead to a compensatory release of epinephrine and norepinephrine, potentially leading to rebound hypertension and, in turn, a potential increase in ICP. Additionally, patients can have a variable response to BP lowering as well as develop tachyphylaxis [6]. Existing literature recommends using hydralazine with caution in patients with ICH due to the potential increase of ICP. However, this recommendation relies on a case series published in 1975, which describes only three patients [7]. Two of the patients included were following a diagnosis of hemorrhagic stroke and one was after a traumatic brain injury. All three patients received between 12.5 mg and 15.8 mg of IV hydralazine. The patient’s intraventricular pressure was evaluated at two points following the ICH and all three patients experienced paradoxical increases in mean intraventricular pressure. The authors report an average increase in ICP of 110% that preceded the decrease in systemic BP following administration of hydralazine [7].

Given that the available published evidence is so limited, more research is necessary to fully establish the safety of IV hydralazine for BP management in patients with ICH. The purpose of this study is to compare the safety and efficacy on ICP of IV hydralazine to IV labetalol in patients with ICH. The stated null hypothesis is there is no difference in ICP elevation or management of ICP when using hydralazine compared to labetalol.

## Materials and methods

This is a retrospective chart review of patients admitted to a 550-bed level I trauma center and primary stroke center in Northeast Ohio between September 2015 and September 2021. Patients were identified through a search of surgery and trauma registry records to identify patients with records of placement of an ICP monitor or external ventricular drain (EVD) with documented ICP measurements. Electronic medical charts were reviewed for the identified patients and screened for inclusion and exclusion criteria and to collect pertinent patient data.

Patients were included in the analysis if they were 18 years or older and had an indication for ICP monitoring. Potential indications included subdural hematoma (SDH), epidural hematoma (EDH), subarachnoid hemorrhage (SAH), spontaneous intracerebral hemorrhage, aneurysmal subarachnoid hemorrhage (aSAH), and diffuse axonal injury (DAI). Patients required documented ICP measurements within zero to 80 minutes prior to and after medication administration to be included. Finally, patients needed to have elevated BP requiring pharmacologic intervention with either IV hydralazine or IV labetalol. Patients were excluded from the analysis if they were younger than 18 years or had no ICP monitor data available. Single administration data points were excluded if multiple as needed antihypertensive agents were administered within the same 80-minute period or a new scheduled antihypertensive agent was initiated within the same 80-minute period. An 80-minute period was selected for data collection and outcome reporting to include the entire time period of the expected onset of action of hydralazine [6].

The primary outcome of this analysis was the mean difference in ICP within zero to 80 minutes comparing IV hydralazine to IV labetalol. Secondary outcomes included differences in clinical interventions required for elevated ICP within zero to 80 minutes of medication administration. Clinical interventions included a bolus dose of hypertonic saline, a bolus dose of mannitol, hyperventilation, additional CSF drainage, additional head of bed elevation, additional opioid or sedative medications, administration of neuromuscular blocking medications, initiation of pentobarbital coma, and emergent need for craniotomy or craniectomy.

Categorical variables were compared using Chi-square test and Fischer’s exact tests. Continuous variables were compared using analysis of variance (ANOVA). A p-value of 0.05 was considered statistically significant. The data is presented using a number (%) or mean with 95% confidence interval (CI) and standard deviation as appropriate.

## Results

During the study period, a total of 73 patients were identified with an ICP monitor or EVD and were screened for inclusion. A total of 27 patients were included in the final analysis, two patients were excluded as they were under the age of 18, 29 patients did not have any eligible administrations of labetalol or hydralazine, 12 patients did not have documented ICP measurements surrounding medication administration, and one patient was pronounced brain dead prior to medication being administered. Of the 27 included patients, three (11.1%) received only hydralazine, 13 (48.1%) patients received only labetalol, and 11 (40.7%) patients received both medications during their stay. Overall, there were 27 (19%) doses of hydralazine and 115 (81%) doses of labetalol.

The included patients were predominantly male (85.2%; n = 23) and had a mean age of 40 years (range 20-97; standard deviation (SD) 18.479). Based on the initial GCS (m = 6.37; range 3-14; SD 3.764), the results indicate a majority of patients classified as having a severe brain injury, which aligns with patients indicated for invasive ICP monitoring devices in this study. Most hemorrhages were traumatic in origin. Complete baseline hemorrhage characteristics are displayed in Table 1.

**Table 1:** Baseline hemorrhage characteristics ICH: intracranial hemorrhage; n: number; SD: standard deviation

Characteristics	Percentage (n)
Traumatic ICH	92.5% (25)
Spontaneous ICH	7.4% (2)
Subdural hematoma	70.4% (19)
Subarachnoid hemorrhage	11.1% (3)
Intraventricular hemorrhage	7.4% (2)
Midbrain hemorrhage	3.7% (1)
Pontine hemorrhage	3.7% (1)
Unspecified ICH	3.7% (1)

The mean difference in ICP between zero and 80 minutes of medication administration was 0.19 mmHg (95% CI: -1.57 to 1.94) for patients given hydralazine and -0.077 mmHg (95% CI: -1.53 to -0.01) for patients administered labetalol, there was no significant difference between groups. When comparing the proportion of patients that had any increase in ICP, hydralazine had 10.3% more instances of an increase than labetalol, which was not significant between groups (odds ratio (OR): 0.661).

When evaluating for clinical interventions for an elevated ICP, the difference between hydralazine and labetalol was less than 5%. There was also no significant difference noted in the number of interventions required per dose. The hydralazine arm required a mean of 1.88 interventions versus per dose and the labetalol group required 1.41 interventions. One patient required a maximum of five interventions following a dose of hydralazine, which prompted additional review. Based on the clinical information provided in the patient’s record, it was likely that hydralazine was administered concomitantly to acutely lower BP in a patient at risk of imminent herniation. No patients included in this study had explicitly documented herniation. See Table 2 and Table 3 for complete results.

**Table 2:** Comparison of hydralazine and labetalol to ICP outcomes ICP: Intracranial pressure; mmHg: millimeters of mercury; SD: standard deviation; n: number

Outcomes	Hydralazine	Labetalol	Significance
Mean difference in ICP, 0-80 minutes	0.190 mmHg (SD 4.438)	-0.077 mmHg (SD 4.066)	p = 0.283
Proportion of patients with any increase in ICP	51.9% (n = 14)	41.6% (n = 47)	p = 0.390
Any intervention required for elevated ICP within 0-80 minutes	29.6% (n = 8)	25.2% (n = 29)	p = 0.633
Mean number of ICP interventions required	1.88 (SD 1.126)	1.41 (SD 0.568)	p = 0.115

**Table 3:** First interventions needed for ICP elevation ICP: Intracranial pressure; n: number

Intervention	Hydralazine% (n)	Labetalol% (n)
No intervention	70.4% (19)	81.9% (86)
Hypertonic saline bolus	3.7% (1)	1.7% (2)
Hypertonic saline infusion	0% (0)	4.3% (5)
Additional opioids/sedatives	22.2% (6)	17.4% (20)
Paralytics	3.67% (1)	1.7% (2)

## Discussion

After an initial hemorrhagic insult to the brain, mass lesions, an increase in cerebral edema, and an increase in the amount of blood in the brain can lead to rising pressure inside the space of the human skull [8]. Brain injury is commonly categorized by the GCS as a bedside modality to assess the degree of patients’ impaired level of consciousness [9]. Out of a total of 15 points, a score of 14-15 is considered a minor injury, a score of 9-13 is considerate moderate, and any score of less than or equal to eight is considered severe [10]. If the GCS is ≤8, it is recommended as a standard of care that ICP monitors be placed for monitoring purposes in various clinical scenarios [11-12]. Management of CPP is achieved through various nonpharmacologic and pharmacologic interventions in order to manipulate the CPP = MAP - ICP equation [13]. The nonpharmacologic options include hyperventilation and manually draining the CSF from the EVD. Hyperventilation, which causes a decrease in the partial pressure of carbon dioxide (PCO2), leads to vasoconstriction, lowering cerebral blood volume and ICP [1]. However, this should only be utilized short-term for acute ICP elevations as it has been shown to exacerbate cerebral ischemia with sustained utilization [1]. The pharmacologic interventions include hyperosmolar therapy, analgesia/sedation, paralytics, and BP agents. Hyperosmolar therapy can be achieved with either hypertonic saline or mannitol [1, 14]. The hypertonic saline can be utilized as either a bolus administration with 3% or 23.4% saline or a 3% continuous infusion with monitoring of sodium levels and serum osmolality. Mannitol and hypertonic saline create an osmolar variant between the vasculature and brain tissue leading to a decrease in inflammation [1,14]. Additionally, analgesic and/or sedative medications can be administered or increased to decrease metabolic demand and thereby decrease ICP. Neuromuscular blockers have also been used to decrease metabolic demand by ceasing all bodily movement. However, due to the morbidity and long-term weakness of paralytics, this is no longer used routinely [2].

Another intervention commonly utilized is BP management. Vasopressors can be initiated to increase the patient’s MAP in hypotensive patients, which will increase the CPP. However, in hypertensive patients, bleeding can be further exacerbated leading to hematoma expansion and ICP elevation [15-16]. Although the timing and goal target systolic continue to be debated, targeting SBPs of <140 mmHg is considered safe in patients with acute intracerebral hemorrhage [17-19]. In order to keep an SBP of <140 mmHg, antihypertensives are recommended to assist in minimizing further bleeding [20]. When IV continuous infusions such as nicardipine and clevidipine are not necessary to maintain BP parameters, labetalol is recommended for this indication as an IV bolus option but is contraindicated in patients with bradycardia [2,5]. Therefore, despite the limited data to show its safety in this patient population, hydralazine is routinely utilized [21].

The concern for rebound ICP elevation was seen in the prior case series [7]. However, we found no statistically significant difference in the primary outcome mean difference in ICP within zero to 80 minutes of administration of hydralazine and labetalol. We also found no significant difference in any of the secondary outcomes including clinical interventions required for an elevated ICP. The authors hoped to compare and evaluate the use of these agents on clinical outcomes, such as documented herniation, hospital and intensive care unit (ICU) length of stay, in-hospital mortality, and discharge disposition. Nonetheless, this was not possible. Given that 40% of the included patients were administered with both hydralazine and labetalol during the study period, it was not feasible to directly compare the impact of the individual agents on patient-centered outcomes.

This study was noted to have several limitations. It is limited in broad clinical application due to the retrospective design of the study, the small sample size, and patients only being identified from a single study site. Although it appears that hydralazine is safe in this population based on our results, more detailed and prospective studies are needed to apply this finding more broadly. An additional limitation to the study was accurately identifying all patients with ICP monitoring during the study period. We were challenged by incomplete surgical records and lack of a reliable way to search for all eligible patients. The majority of the study patients had an ICH of traumatic origin in part because the institution is a level one trauma center that treats a higher population of trauma patients. But additionally, due to incomplete surgical records, a search of the trauma registry was completed in an attempt to increase the sample size. Another limitation to the study was the low percentage of patients receiving hydralazine when compared to labetalol. This may be in part due to the common structure of as needed antihypertensive orders at this institution. Most patients were ordered both hydralazine and labetalol as needed with administration parameters distinguished by heart rate to meet order requirements by The Joint Commission. This resulted in hydralazine being utilized primarily for patients with lower heart rates, which was less common among this patient population. Lastly, the study investigators had hoped to have additional secondary outcomes measured, including patient's length of stay, mortality, and discharge disposition. However, the majority of patients included in the study received both hydralazine and labetalol, making these comparisons unfeasible.

## Conclusions

Existing literature, consisting of one case series, recommends using hydralazine with caution in patients with ICH due to the potential increase of ICP. In the current study, there was no significant difference in mean ICP from zero to 80 minutes following the administration of hydralazine or labetalol. There was also no significant difference in interventions required for elevated ICP management between patients receiving hydralazine and labetalol. It appears that both hydralazine and labetalol can be safely used in this patient population; however, larger studies are needed to confirm these findings.
